# De novo transcriptome analysis shows differential expression of genes in salivary glands of edible bird’s nest producing swiftlets

**DOI:** 10.1186/s12864-017-3861-9

**Published:** 2017-07-03

**Authors:** Q. H. Looi, H. Amin, I. Aini, M. Zuki, A. R. Omar

**Affiliations:** 1 Institute of Bioscience, Universiti Putra Malaysia, 43400 UPM Serdang, Selangor, Malaysia; 2 Faculty of Veterinary Medicine, Universiti Putra Malaysia, 43400 UPM Serdang, Selangor, Malaysia

**Keywords:** *Aerodramus fuciphagus*, *Aerodramus maximus*, *Apus affinis*, Transcriptome, Salivary gland

## Abstract

**Background:**

Edible bird’s nest (EBN), produced from solidified saliva secretions of specific swiftlet species during the breeding season, is one of the most valuable animal by-products in the world. The composition and medicinal benefits of EBN have been extensively studied, however, genomic and transcriptomic studies of the salivary glands of these birds have not been conducted.

**Results:**

The study described the transcriptomes of salivary glands from three swiftlet species (28 samples) generated by RNASeq. A total of 14,835 annotated genes and 428 unmapped genes were cataloged. The current study investigated the genes and pathways that are associated with the development of salivary gland and EBN composition. Differential expression and pathway enrichment analysis indicated that the expression of *CREB3L2* and several signaling pathways involved in salivary gland development, namely, the EGFR, BMP, and MAPK signaling pathways, were up-regulated in swiftlets producing white EBN (*Aerodramus fuciphagus*) and black EBN (*Aerodramus maximus*) compared with non-EBN-producing swiftlets (*Apus affinis*). Furthermore, *MGAT*, an essential gene for the biosynthesis of N-acetylneuraminic acid (sialic acid), was highly expressed in both white- and black-nest swiftlets compared to non-EBN-producing swiftlets. Interspecies comparison between *Aerodramus fuciphagus* and *Aerodramus maximus* indicated that the genes involved in N-acetylneuraminic and fatty acid synthesis were up-regulated in *Aerodramus fuciphagus,* while alanine and aspartate synthesis pathways were up-regulated in *Aerodramus maximus.* Furthermore, gender-based analysis revealed that N-glycan trimming pathway was significantly up-regulated in male *Aerodramus fuciphagus* from its natural habitat (cave) compared to their female counterpart.

**Conclusions:**

Transcriptomic analysis of salivary glands of different swiftlet species reveal differential expressions of candidate genes that are involved in salivary gland development and in the biosynthesis of various bioactive compounds found in EBN.

**Electronic supplementary material:**

The online version of this article (doi:10.1186/s12864-017-3861-9) contains supplementary material, which is available to authorized users.

## Background

Swiftlets are small, aerial, insectivorous birds, and are widely distributed; they extend from the Western Indian Ocean to the Pacific Ocean [1, 2]. The swiftlet species roost in caves or in a cave-like, man-made environments called swiftlet houses [3]. During the breeding season, both male and female swiftlets of the *Aerodramus* species collaborate to produce highly valuable edible bird’s nest (EBN) from saliva secretions generated from their salivary glands [4, 5]. Hence, the salivary glands of these swiftlets are relatively bigger during the breeding season compared to other seasons [6]. A study on the anatomy of swiftlet report that the sublingual glands of swiftlet enlarge tremendously during the breeding season, i.e. from 2.5 mg to 160 mg, while fledgling birds were found to have small, non-secretory salivary glands [6, 7]. Such dramatic changes in the gland size and weight are probably associated with the proliferation and differentiation of specialized cells, presumably stem cells, which control the development of the glands during breeding season [8]. However, no significant enlargement of salivary glands was observed in non-EBN-producing swiftlets (*Apus* species) which produce nests that primarily comprise of dried grass [4].

EBN is a luxurious delicacy, and has been used in Traditional Chinese Medicine for hundreds of years; it is known as one of the world’s most valuable animal products [4, 7]. Nests from the white-nest swiftlet (*Aerodramus fuciphagus*) and the black-nest swiftlet (*Aerodramus maximus*) are harvested for commercial purposes. Compared with black-nests (*A. maximus*), white-nests from *A. fuciphagus* are premium grade as they comprise solely of solidified saliva secretions and contain high concentrations of N-acetylneuraminic acid (sialic acid) and epidermal growth factor (EGF) [4, 7]. The current market value of EBN varies from USD 100 to USD 2000 per kilogram, depending on the shape, size, origin, and color of the nest [9]. Over the decades, the composition of EBN has been studied by several researchers [10], but limited studies were conducted on the swiftlets’ salivary glands, which produce saliva that solidifies and forms the EBN consumed by humans, either for its medicinal properties or as a delicacy. Swiftlets complete the construction of their nests using saliva secretions in approximately 35 days, and each nest weighs between 8 to 12 g. The composition of EBN consists mainly of protein (62–63%) and carbohydrates (25–27%) [4], followed by lipids (0.14–1.28%), and ash (2.10%), though the contents of EBN are influenced by seasonal variations and breeding sites [11]. Several bioactive compounds with health-promoting effects were identified in EBN, such as glucosamine, lactoferrin, sialic acid, amino acids, fatty acids, triacylglycerol, vitamins, minerals, and other anti-oxidant molecules [12]. Despite efforts to investigate the composition of EBN and its medicinal properties, little is known regarding the genes that are transcriptionally active in the salivary glands of swiftlets.

Genomic characterization of avian species has been proven to be of importance for both socio-economic and ecological reasons as many avian species are vital to agricultural and poultry industries. Hence, the genome sequences of several avian species, such as that of chickens, pigeons and turkeys, have been investigated over the years [13–15]. However, studies on genes or the genome of swiftlets are scarce. As of February 2017, there were approximately 850 sequences under *Aerodramus* spp. in the nucleotide database of NCBI, with *Aerodramus fuciphagus* being the most extensively studied species. The absence of an established swiftlet genome has hindered many fundamental studies on salivary gland development and saliva secretion during EBN construction.

Traditionally, RNA sequencing projects require tedious laboratory manipulation and are time-consuming. However, current advancements in next-generation sequencing (NGS) techniques have provided researchers with cost- and time-effective platforms for transcriptomic analyses [16–18]. In the current study, we performed de novo transcriptome sequencing of EBN-producing swiftlets (*Aerodramus fuciphagus* and *Aerodramus maximus*) and non-EBN-producing swiftlets (*Apus affinis*) to generate individual reference transcripts for each species. The generated reference transcripts were further analyzed for gene annotation, pathway enrichment, and differential gene expression studies. The transcriptome profiles of the salivary glands of the *Aerodramus* species and the *Apus affinis* showed us a differential expression of genes that are important in saliva secretion and the composition of EBN during nest construction.

## Methods

### Animal ethics and sampling

RNA sequencing libraries were derived from the salivary glands of *Aerodramus fuciphagus* (*A. fuciphagus*), *Aerodramus maximus* (*A. maximus*), and *Apus affinis* (Additional file 1: Table S1). A total of 16 *Aerodramus fuciphagus* samples (eight from a natural habitat, i.e. caves, and eight from a man-made habitat, i.e. houses), 8 *Aerodramus maximus* samples from a cave, and 2 *Apus affinis* samples were collected from various locations (Additional file 1: Table S1). Salivary gland samples of *A. maximus* from man-made houses were not included in this study as farmers rarely breed *A. maximus* due to the low value of its nests compared with *A. fuciphagus*. Upon necropsy, the sublingual salivary glands were removed, weighed, and then stored in RNAlater® solution (Ambion, USA) at −80 °C for RNA extraction. Only well-developed salivary glands (0.06–0.10 g) and partially developed salivary glands (0.01–0.05 g) from adult birds were considered for the RNA sequencing (Additional file 2: Table S2). This sampling strategy was adopted to obtain unbiased and comprehensive transcriptomic profiles, because during the breeding season swiftlets have both types of salivary glands.

### Species identification

Identification of the species of the sampled swiftlets was carried out based on the sequencing of the cytochrome-b gene, as described in a previous study [1].

### RNA isolation

Approximately, 30 mg of salivary gland tissues were individually homogenized (TissueRuptor, Qiagen) before RNA extraction. Total RNA was extracted using the RNeasy® Plus kit (Qiagen, German) according to the manufacturer’s protocol. The extracted RNA was stored at −80 °C until further use. The integrity and purity of the extracted RNA were checked using a spectrophotometer (Eppendorf, Germany) and a bioanalyzer (Agilent, US).

### Sequencing and data analysis

The sequencing was performed on an Illumina HiSeq 2000 system (100 bp pair-end read) using Illumina TruSeq Cluster V3 flow cells, and a TruSeq SBS Kit V3 (Illumina, US) according to the manufacturer’s specifications. Each sample was sequenced individually to generate individual sequencing reads. Quality control of the RNA-Seq data was performed using FastQC. A total of 5 parameters were checked during quality control analysis, namely sequence quality scores (Phred Score), base sequence content, base and sequence GC content, sequence length distribution, sequence duplication level, and kmer content. The fastQ file adapters were removed using Cutadapt [19] and reads with a Phred score below 20 were removed using the FASTX-Toolkit. The cleaned reads were deposited in the NCBI Sequence Read Archive (SRA) under accession number SRX1640191-SRX1640203 and SRX1640246-SRX1640253. Raw reads were assembled using SOAPdenovo-Trans software with the default parameters [20]. Prior to the assembly, raw reads generated from each library were concatenated, and redundant reads were removed using Fastq-MCF software in each sampling group [21]. A total of 4 reference transcripts for *Aerodramus fuciphagus* from the cave (AFC), *Aerodramus fuciphagus* from the man-made house (AFM), *Aerodramus maximus* from the cave (AMC), and *Apus affinis* (AA) were generated. Core Eukaryotic Genes Mapping Approach (CEGMA) analysis was performed to validate the completeness and accuracy of the transcript assembly [22] (Additional file 3: Figure S1).

### Gene, functional and pathway annotation

Raw data from the four groups were concatenated to generate a non-redundant reference assembly using the CAP3 program for gene annotation and differential expression analysis [23]. A non-redundant reference transcript was used for gene prediction by the AUGUSTUS program (University of Greifswald, Germany) [24]. Gene predictions were retrieved in a general feature file (GFF) format and used for downstream annotation and expression analyses. Functional annotation was performed using the Blast2GO tool [25]. All transcript sequences were aligned to sequences, in order of priority, using BLASTX (cut-off E-value of <0.0005), in the following databases: NR (NCBI database), InterPro database, UniProt database, Pfam database, Kyoto Encyclopedia of Genes and Genome database (KEGG), and WikiPathways. The highest ranks in the blast results were used to identify the coding region of the transcript sequences. Gene Ontology (GO) annotation was performed using Blast2Go software v2.5.0. The BLASTX cut-off value was set to 1 × 10^6^. Each annotated sequence was assigned to detailed GO terms. Proteins were further classified into three main GO categories, namely molecular function, cellular component, and biological process, using Web Gene Ontology Annotation Plot (WEGO) (BGI Americs, UK) [26]. KEGG pathway analysis was conducted using the KEGG Automatic Annotation Server (KASS) available at http://www.genome.jp/tools/kaas/. GO is a hierarchical description of gene function that classifies genes based on known or predicted functions in model organisms [27]. GO terms allow a general assessment of the annotation and functional roles of an individual gene. However, GO annotation that is based on sequence similarity alone may result in an over-assignment of GO terms to genes that are functionally diverged [28]. Thus, this study investigated the large-scale pathway patterns revealed by these functional annotations.

### Gene expression analysis

Raw reads in the fastQ format from Illumina sequencing were mapped against the reference assembly generated by the TopHat v2.0.10 software [29] to provide a uniform template to calculate gene expression levels in various groups using the Cuffdiff application of the software. To compare expression analysis among samples, the BAM files from TopHat and the predicted GFF files from AUGUSTUS were used as inputs for Cuffdiff v2.1.1 software, and then normalized with the FPKM (fragments per kb per million reads) method to identify the differentially expressed genes among the samples [16]. The following comparisons of gene expression profiles were made: a) EBN-producing swiftlets and non-EBN-producing swiftlets, (b) interspecies comparisons among EBN-producing swiftlets from the cave (AFC and AMC), and lastly, (c) habitat comparisons between AFC and AFM. Furthermore, analyses were also performed to determine the relationships between gender and expression profiles of EBN-producing swiftlets. To evaluate the significantly regulated pathways, the list of differentially expressed genes with a log2 fold change >2 and a *p* < 0.05 was keyed into the Enricher tool, and the gene set was annotated for possibly affected pathways using the KEGG, Reactome, Biocarta and PANTHER databases.

### Validation of expression profile

A total of 21 well-developed salivary gland samples with five samples previously analyzed by NGS transcriptomic analysis were selected for the gene expression validation study. A total of 36 genes of potential importance in salivary gland development and EBN composition were selected for validation from the total of 14,835 genes detected by NGS expression profiles using the NanoString nCounter® gene expression assay. Total RNA was extracted using the RNeasy® Plus kit (Qiagen, Germany) according to the standard protocol. The array contained four internal positive control probes and five negative controls for data normalization. Seven housekeeping genes were selected based on expression profile analysis to determine the appropriate thresholds for measuring significant hybridization signals over the background. After pre-processing and normalization, log2-transformed RNA-Seq and nCounter data from 36 genes were compared, and the concordance of the data from both technologies were evaluated.

## Results

### Overview of the swiftlets’ transcriptome landscape

After removing the amplification adapters and the ambiguous reads, we generated a total of 17.4 million, 17.7 million, 19.6 million, and 15.1 million clean reads for AFC, AFM, AMC, and AA, respectively (Table 1). The results showed that 99% of the sequenced reads passed quality control with Phred scores of more than 20, indicating that the error rate of the sequencing process was less than 0.01%. SOAP*denovo-Trans* software was used to perform de novo assembly and generated 47,596 (AFC), 55,331 (AFM), 40,330 (AMC), and 44,145 (AA) contigs; the average contig size exceeded 200 bp for all four libraries (Table 3). More than 65% of the reads could be mapped back to the reference transcripts in all four experimental groups. Furthermore, for all four groups, more than 96% of the genes were longer than 100 bp, more than 20% of the genes were 500 to 1000 bp, 11% of the genes were longer than 1000 bp, and 0.01% of the genes for AFC, AFM, and AA exceeded 10 kbp (Table 1). CEGMA analysis was used to analyze the completeness of the assembled transcript sequences. A total of 142 (57.66%) Core Eukaryotic Genes (CEGs) were identified as full-length, whereas a total of 185 (74.6%) core genes were retrieved as partial CEGs (Additional file 4: Table S3).

**Table 1:** Overview of the sequencing and assembly

Sequencing parameters	Species and location			
	AFC	AFM	AMC	AA
Contig (n)	47,596	55,331	40,330	44,145
Contig N50 size	252	269	256	244
Total length of contig	10,865,350	12,946,212	9,192,670	9,807,398
Scaffolds (n)	28,471	32,337	25,185	28,051
Scaffold N50 size	1521	1560	1362	1143
Maximum length of scaffold	15,860	15,605	10,769	15,348
Minimum length of scaffold	100	100	100	100
Total length of scaffolds	13,284,125	15,888,505	11,131,881	11,571,111
GC percentage	46.72%	44.30%	47.94%	50.73%

### Functional annotation and classification of the assembled Unigenes

**Fig. 1:**
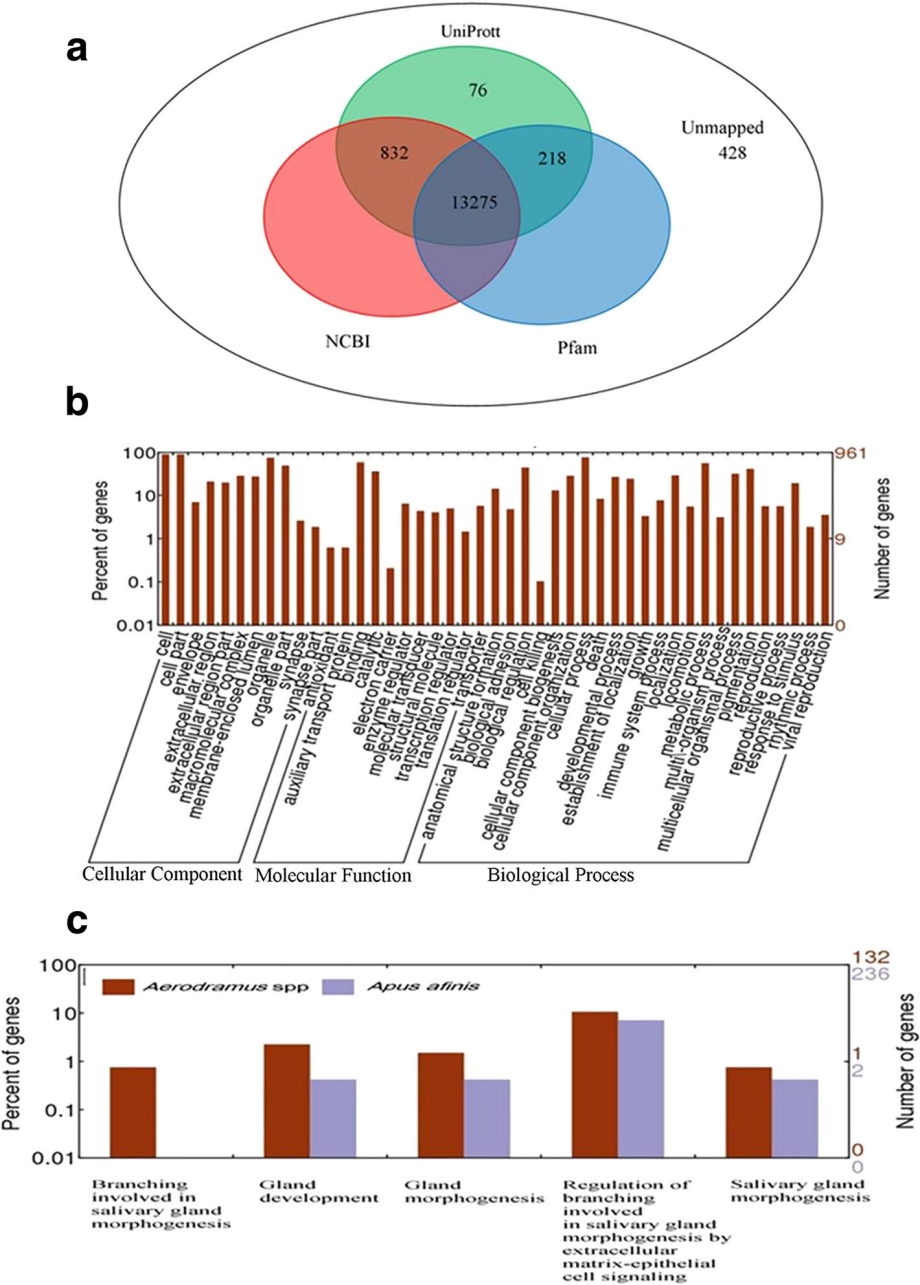
Gene annotation and gene ontology of swiftlet transcriptome. **a** Venn diagram showing unique and shared proteins following annotation based on Non-redundant, InterProsca and Pfam databases. **b** There are 14,107 proteins assigned to the cellular component, biological process and molecular function by Gene Ontology classification system. The y-axis indicates the percentage of genes for each category, and the y-axis on the right side shows the number of genes. **c** The number of genes involved in salivary gland development between *Apus affinis* and *Aerodramus* species. The y-axis indicates the percentage of genes for each category, and the y-axis on the right side shows the number of genes

The generated, non-redundant, reference transcript was used for gene predictions by AUGUSTUS and a total of 14,835 genes were predicted. The functional annotation of genes was first performed using the BLASTx function in the Blast2GO tool against the non-redundant (NR) protein database from NCBI and the UniProt database with an E-value of <0.0005. Out of 14,835 high-quality genes, 14,107 genes (95.51%) had a minimum of one significant match to an existing gene model in the Blast2Go database and mapped to 7028 different protein IDs in the UniProt database. Pfam was used to further improve the functional annotation process. In total, 13,493 out of 14,835 genes (97.81%) were mapped to the known functional domain in the Pfam database using multiple sequence alignments and hidden Markov models (HMMs). However, 428 genes (2.89%) were not identified in any of the databases and were classified as hypothetical proteins (Figure 1a). Gene ontology analysis indicated that a total of 14,107 genes were categorized into 49 GO clusters based on three main categories, namely cellular components, molecular function, and biological processes. The molecular function category consisted of 11 GO groups, the largest group was that of binding (60.1%), and the cellular component category consisted of 15 GO groups; the largest group was that of cell/cell parts (90.6%). Lastly, the biological processes category consisted of 23 GO groups; the largest group was that of cellular process (77.4%) (Figure 1b). Further gene ontology enrichment analysis showed that more genes were involved in salivary gland morphogenesis and regulation in *Aerodramus* than in *Apus affinis* (Figure 1c).

### Gene expression analysis

**Fig. 2:**
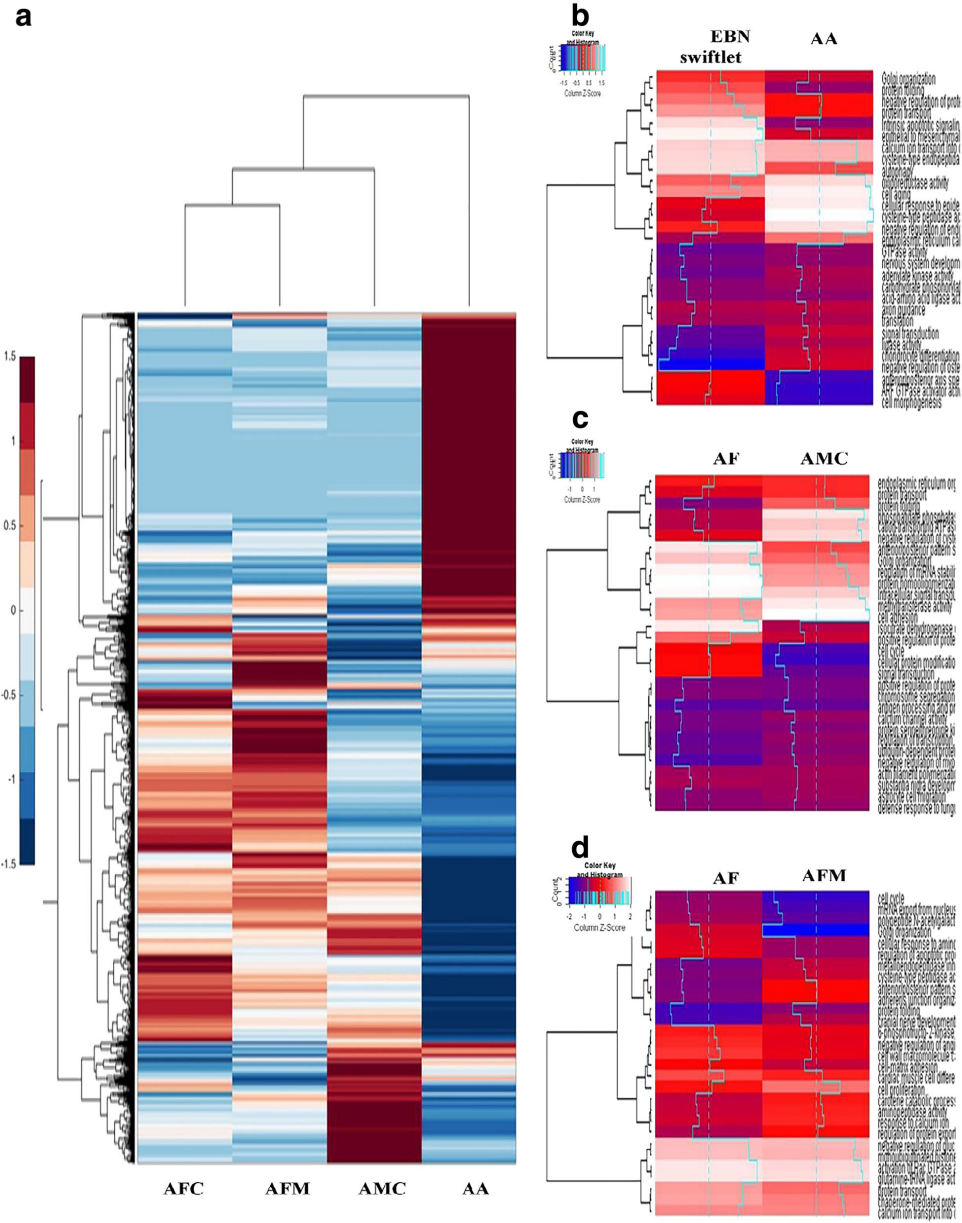
Gene expression profiles between different swiftlets species. **a** Comparison of relative gene expression between AFC, AFM, AMC and AA. **b** Top-10 differential expressed genes between EBN producing swiftlets (*A. fuciphagus* and *A. maximus*) and non-EBN producing swiftlets (AA). **c** Top-10 differential expressed genes between *A. fuciphagus* and *A. maximus* from natural habitat (cave). Genes were identified using a combination of ANOVA with Bonferroni-corrected ≤0.05. **d** Top-10 differential expressed genes between *A. fuciphagus* from natural habitat (cave) and man-made habitat (house). Genes were identified using a combination of ANOVA with Bonferroni-corrected ≤0.05. Expression data were Z-score standardized per gene

Data from EBN-producing swiftlets (AFC, AFM, and AMC) was compared to data from non-EBN-producing swiftlets (AA); interspecies comparisons of data from EBN-producing swiftlets from the cave (AFC and AMC), and comparisons of white-nest producing swiftlets from two different habitats (AFC and AFM) (Figure 2). In addition, the effects of gender on gene expression patterns were also studied for EBN-producing swiftlets. As expected, the greatest differential gene expression profile was observed between EBN-producing swiftlets (AFC, AFM, and AMC) and non-EBN-producing swiftlets (AA). Comparisons between the data from EBN-producing swiftlets and non-EBN-producing swiftlets showed that a total of 2037 genes were significantly up-regulated and 1450 genes were significantly down-regulated. An interspecies comparison between AFC and AMC from similar habitats showed a fewer number of differentially regulated genes; only expressions of 124 genes were up-regulated and 118 genes were down-regulated. Lastly, expression profiles between AFC and AFM only identified 16 up-regulated genes and 32 down-regulated genes out of 14,835 annotated genes. Analysis of male and female samples indicated that AFM have the highest number of regulated genes, whereby a total of 180 genes were significantly up-regulated in male samples and 37 genes in female samples (Table 2). However, only 102 genes and 45 genes were significantly up-regulated in AFC and AMC samples, respectively (Table 2). Overall analysis of log2-fold change data suggested that 98% of the significantly regulated genes have less than 4 folds different.

**Table 2:** Number of up-regulated genes of male and female swiftlet salivary glands

Gender of the swiftlets	Sampling groups		
	AFC	AFM	AMC
Male^a^	46	180	20
Female	56	37	25

^a^Samples from male swiftlets were used as baseline for differential expression analysis

### Pathway prediction and enrichment analysis

A total of 14,835 annotated genes were exported to the Enricher tool for pathway enrichment analysis against several publicly available databases. The results indicated a total of 41, 75, 11, 69, and 34 pathways from KEGG, WIKI pathway, Reactome, Biocarta, and PANTHER databases, respectively. All genes that were significantly regulated with a q-value less than or equal to 0.05 and a log2-fold change of more than two were identified and subjected to pathways enrichment analysis.

Pathway enrichment analysis between EBN and non-EBN-producing swiftlets indicated that a total of 341 pathways were up-regulated in EBN-producing swiftlet salivary glands, with a majority of the pathways involved in mRNA processing (15.5%), protein export (14.4%), and squamous cell TarBase (9.1%), while 328 pathways were up-regulated in AA samples. The top 3 up-regulated pathways identified in AA were associated with translation factors (27.4%), mRNA processing (10.9%) and spliceosomes (8.8%). Further analysis showed a total of 152 and 120 up-regulated pathways detected among AFC and AMC samples, respectively. The majority of the up-regulation in AFC samples involved the PI3K-Akt signaling pathway (46%), ribosome biogenesis (12.5%), and cardiac muscle contraction (6.5%). Meanwhile, analysis of AMC samples showed that the top three up-regulated pathways were the Ras signaling pathway (38%), the MAPK signaling pathway (29%), and the proteasome pathway (20%). As expected, comparisons between AFM and AFC showed only 34 and 7 pathways that were significantly up-regulated in AFM and AFC samples, respectively. The majority of the detected pathways in AFM samples were associated with galactose metabolism (38%), endocytosis (15%), and oxidative phosphorylation (15%), while the top three up-regulated pathways in AFC samples were mitochondrial fatty acid oxidation (43%), RNA transport (29%), and proteoglycan (29%) (Table 3).

**Table 3:** Pathway enrichment analysis of up-regulated pathways in swiftlet salivary glands

AA versus Aerodramus species	Number of pathways up-regulated	Top 3 regulated pathway
AA	328	- Translation factors (27.4%)
		- mRNA processing (10.9%)
		- Spliceosome (8.8%)
Aerodramus species	341	- mRNA processing (15.5%)
		- Protein export (14.4%)
		- Squamous cell TarBase (9.1%)
AFC versus AMC	Number of pathways up-regulated	Top 3 regulated pathway
AFC	152	- PI3K-Akt signaling pathway (46%)
		- Ribosome biogenesis in eukaryotes (12.5%)
		- Cardiac muscle contraction (6.5%)
AMC	120	- Ras signalling pathway (38%)
		- MAPK signalling pathway (29%)
		- Proteasome (20%)
AFM versus AFC	Number of pathways up-regulated	Top 3 regulated pathway
AFM	34	- Galactose metabolism (38%)
		- Endocytosis (15%)
		- Oxidative phosphorylation (15%)
AFC	7	- Mitochondrial fatty acid oxidation (43%)
		- RNA transport (29%)
		- Proteoglycan (29%)
Gender comparison		
AFC	Number of pathways up-regulated	Top 3 regulated pathway
Female	34	-DNA repair (61%)
		-Circadian clock (20%)
		-Cell cycle (12%)
Male	25	-Mitochondrial protein (40%)
		-Peptide hormone metabolism (23%)
		-Muscle contraction (15%)
AFM	Number of pathways up-regulated	Top 3 regulated pathway
Female	0	-NA
Male	15	-Transmembrane transport of small molecule (17.5%)
		-Protein metabolism (5.8%)
		-Signal transduction (5.1%)
AMC	Number of pathways up-regulated	Top 3 regulated pathway
Female	13	-Organelle biogenesis and maintenance (23%)
		-Muscle contraction (17%)
		-Axon guidance (9%)
Male	5	-Vesicle-mediated transport (38%)
		-Extracellular matrix transport (29%)
		-Signal transduction (23%)

*AFC Aerodramus fuciphagus* from cave

*AFM Aerodramus fuciphagus* from man-made house

*AMC Aerodramus maximus* from cave

*AA Apus affinis*

*NA* No regulated pathways with *p*-value <0.05 was identified

Gender based analysis showed that a total of 34 pathways were significantly up-regulated in female AFC samples, and the majority of these pathways were associated with DNA repair (61%), circadian clock (20%), and cell cycle (12%). Furthermore, 25 pathways were up-regulated in male samples, and 40% of the identified pathways were involved in mitochondrial protein synthesis. Analysis of AFM data did not indicate significant regulated pathways in female samples, whereas 15 pathways were significantly up-regulated in male samples. The majority of the detected pathways were associated with molecule transport (17.5%), protein metabolism (5.8%), and signal transduction (5.1%). Lastly, a total of 13 and 5 pathways were up-regulated in female and male samples from AMC, respectively. Majority of the regulated pathways from female samples were related to organelle biogenesis (23%), muscle contraction (17%), and axon guidance (9%), while the top three regulated pathways in male samples were vesicle-mediated transport (38%), extracellular matrix transport (29%), and signal transduction (23%) (Table 3).

A total of 24 genes of interest, that are associated with salivary gland development and EBN composition, were identified for further discussion. These genes are involved in salivary gland development, the epidermal growth factor receptor (EGFR) signaling pathway, the bone morphogenetic protein (BMP) signaling pathway, the mitogen-activated protein kinases (MAPK) signaling pathway, the fatty acid synthesis pathway, the N-glycan trimming in endoplasmic reticulum, and the N-glycan biosynthesis pathway (Table 4).

**Table 4:** Expression profiles of genes of potential importance in salivary gland development and EBN compositions

Gene function	Gene	AFM	AFC	AMC	AA
Salivary development	*CREB3L2*	65.608	76.666	51.669	0.000
	*SEC63*	36.723	34.793	50.081	5.926
	*SEC24A*	15.763	15.414	19.240	19.20
	*SAR1*	38.916	23.855	16.996	6.934
AA versus Aerodramus species					
Gene function	Gene	AA	Aerodramus spp	log2	q-value
EGFR signalling pathway	*ARF4*	0.683	48.954	6.162	0.026
	*BCAR1*	6.407	51.836	3.016	0.003
	*CAV1*	0.695	50.207	6.174	0.026
	*CEBPA*	8.626	49.820	2.529	0.018
	*ELF3*	6.816	603.607	6.468	0.001
	*EPS15*	0.198	10.035	5.657	0.026
	*EPS8*	0.754	13.668	4.178	0.026
	*JUN*	0.411	11.504	4.803	0.021
	*PLEC*	6.047	31.197	2.367	0.005
	*RAB5A*	17.934	47.435	1.403	0.046
	*REPS1*	6.795	21.529	1.663	0.040
	*ZPR1*	1.079	13.242	3.617	0.001
	*VAV2*	49.553	239.912	2.275	0.011
	*STAT3*	2.453	53.308	4.441	0.001
N-glycan biosynthesis	*ALG2*	6.546	90.860	3.794	0.001
	*MGAT1*	4.617	91.067	4.301	0.004
	*RPN1*	66.407	552.366	3.056	0.003
	*FUT8*	13.173	84.076	2.674	0.003
	*RPN2*	1.069	71.026	6.053	0.001
AMC versus AFC					
Gene function	Gene	AMC	AFC	log2	q-value
EGFR signalling pathway	*JUND*	4.911	39.541	3.009	0.001
	*HDAC*	1.357	580.771	8.740	0.001
	*BCAR*	7.776	200.736	4.690	0.001
N-glycan biosynthesis	MGAT	20.278	256.555	3.661	0.046
Alanine and Aspartate metabolism	*GOT1*	226.741	7.0263	−5.012	0.000
	*PDHB*	894.023	135.641	−2.721	0.000
AFM versus AFC					
Gene function	Gene	AFM	AFC	log2	q-value
MAPK signalling pathway	*HMGN1*	23,486.800	2685.98	−3.128	0.001
BMP signalling pathway	*TOB2*	20.463	0.959	−4.414	0.001
Fatty acid synthesis	*ACADVL*	10.909	50.803	2.219	0.001
Gender comparison of AFC					
Gene function	Gene	Male	Female	log2	q-value
N-glycan trimming	*EDEM2*	3.951	0.359	3.459	0.008

*AFC Aerodramus fuciphagus* from cave

*AFM Aerodramus fuciphagus* from man-made house

*AMC Aerodramus maximus* from cave

*AA Apus affinis*

### NanoString nCounter^®^ Gene expression assay

A total of 36 genes associated with salivary gland development and EBN compositions were analyzed using the NanoString nCounter® gene expression assay. All the analyzed genes showed a normal distribution of log2 expression values with a median value of 10.5-folds compared with the negative control. Out of these genes, a total of 33 genes showed similar expression patterns as detected by NGS-based transcriptomic analysis, and three genes, namely *PDHB*, *PRMT5* (both involved in amino acid metabolism), and *HK1* (involved in sugar metabolism) showed contradicting results (Table 5). A Pearson correlation was calculated based on normalized, log2-transformed gene expression values from the nCounter data and the RNA-Seq FPKM data of the 36 genes. The mean correlation was 0.54 with a 95% bootstrap confidence interval.

**Table 5:** Expression profiles comparison between NGS transcriptome profiling and NanoString nCounter^®^ Gene Expression Assay

		Illumina RNA-Seq			NanoString Assay		
Gene	Pathway involved	AFM	AFC	log2	AFM	AFC	log2
*ANXA7*	Gland morphology	1.89	35.49	4.23	951.94	1990.60	2.09
*PRMT5*	Amino phosphate metabolism	3.41	103.85	4.92	174.59	162.56	−1.07
*HK1*	Amino sugar metabolism	59,911.80	11,492.50	−2.38	724.05	926.69	1.28
		AMC	AFC	log2	AMC	AFC	log2
*MGAT*	N-glycan biosynthesis	20.27	256.55	3.66	1337.15	2571.24	1.92
*HADHB*	Fatty acid synthesis	7.72	53.47	2.79	2300.80	2619.53	1.14
*ARF1*	Vesicle biogenesis	17.24	73.10	2.08	5540.25	7430.06	1.34
*RAB5*		0.42	35.24	6.38	2276.53	3340.04	1.47
		AA	EBN producing swiftlet	log2	AA	EBN producing swiftlet	log2
*CBPD*	Vesicle biogenesis	8.93	63.31	2.82	10,631.67	11,824.94	1.11
*MGT*		20.27	256.55	3.66	1337.15	2571.24	1.92
*PDHB*	Alanine and aspartate metabolism	894.02	135.64	−2.72	1709.22	2106.15	1.23
*ARF4*	EGFR signalling pathway	0.68	48.95	6.16	3188.13	6970.01	2.19
*BCAR1*		6.40	51.83	3.01	275.97	777.42	2.82
*CAV1*		0.69	50.20	6.17	3476.29	5640.91	1.62
*CEBPA*		8.62	49.82	2.52	1874.12	2127.62	1.14
*ELF3*		6.81	603.60	6.46	2553.18	2836.8	1.11
*EPS15*		0.19	10.03	5.65	1242.43	1915.74	1.54
*EPS8*		0.75	13.66	4.17	1921.08	2849.26	1.48
*JUN*		0.41	11.50	4.80	2756.44	2784.66	1.01
*REPS1*		6.79	21.52	1.66	1148.45	1440.29	1.25
*STAT3*		2.45	53.38	4.44	985.67	1713.33	1.74
*VAV2*		49.55	239.91	2.27	68.78	554.00	8.06
*ZPR1*		1.07	13.24	3.61	1217.42	2078.00	1.71
*ACAD*	Fatty acid synthesis	1.52	25.48	4.05	1778.95	2282.97	1.28
*ALG2*	N-glycan biosynthesis	6.54	90.86	3.79	952.67	1376.55	1.44
*MGAT1*		4.61	91.06	4.30	66.28	1089.17	16.43
*RPN1*		66.40	552.36	3.05	6806.71	9113.06	1.34
*FUT8*		13.17	84.07	2.67	467.63	2465.45	5.27
*RPN2*		1.06	71.02	6.05	2570.82	3420.57	1.33
*G6PD*	Glutathione synthesis	0.18	46.66	8.00	85.54	209.11	2.44
*RARB*	Vitamin A and carotenoid synthesis	11.84	49.92	2.07	1182.35	1190.05	1.01
*RARG*		8.23	296.86	5.17	527.79	1050.6	1.99
*RBP2*		12.19	45.13	1.88	943.37	1745.05	1.85
*RDH10*		0.86	10.44	3.59	4869.85	7627.12	1.57
*RDH12*		37.25	100.33	1.42	280.34	790.51	2.82
*ILVBL*		4.42	87.32	4.30	5.93	347.25	58.55

*AFC Aerodramus fuciphagus* from cave

*AFM Aerodramus fuciphagus* from man-made house

*AMC Aerodramus maximus* from cave

*AA Apus affinis*

## Discussion

### Characterization of the swiftlet transcriptome

With the advancements in sequencing technologies, the genomes of several avian species, including Peking ducks [30], passenger pigeons [15], and vultures [31], have been sequenced and characterized, whereby this inclusion has accelerated the research on functional genomics and improved our understanding of the genetic regulation of important traits. However, for some non-model organisms and minor species, it is not feasible to perform whole genome sequencing because of the expensive cost. Sequencing based on NGS technology has provided an opportunity to use a transcriptomic approach to study mechanisms that control certain traits in non-model organisms [32]. It is generally recognized that EBN is one of the most expensive animal-based products; it is often known as the “caviar of the East” [4]. Nevertheless, the transcriptomic profiles of swiftlets have not been established. The current study focused on de novo RNA sequencing of the salivary glands of both EBN-producing swiftlets and non-EBN-producing swiftlets.

The transcriptomic sequencing was completed using the Illumina Genome Analyzer system platform, HiSeq2000, with a 100 bp paired-end read. A paired-end sequencing protocol was selected for this study because it is critical for the assembly of short (<100 bp) reads and the de novo sequencing approach [33]. Paired-end sequencing allows the assembly of localized regions that are repetitive, as reads that derived from a specific localized copy of a repeat can often be inferred by the placement of their mate-pair reads in unique, flanking sequences [34]. Furthermore, with short reads, the advantages of a paired-end approach are accentuated and this approach is frequently used in several short-read assemblers, including SOAPdenovo [35] that takes advantage of the deBruijn graph calculation in its reads assembly [36]. Using pair-end sequencing, a total of four reference transcripts were generated in this study which showed similar assembly profiles of samples from swiftlets’ salivary glands compared with previous de novo studies of other small avian species, such as the great tit (*Parus major*) and the songbird (*Junco hyemalis*) with 40,000 to 50,000 contigs and 63% to 90% of reads assembled, respectively [37, 38]. This finding indicated that the experimental design, library preparation, sequencing depth, and the number of replicates adopted by the current study were appropriate for various biological function analyses. Individual gene expression analysis, with little or no consideration on pathway effects, often reveals limited information about functional pathways, which are the key factor to a fundamental understanding of the biological systems [39]. Therefore, the analysis of the current study does not select the gene of interest based on individual gene expression profiles, but it focuses on important functional, biological pathways that are significantly up- or down-regulated to provide a comprehensive comparison between different swiftlet species. Salivary gland-specific transcriptomic profiles were examined based on gene expression patterns and pathway enrichment analysis. As expected, the results suggest that the comparison of transcriptomic profiles between non-EBN- and EBN-producing swiftlet salivary glands showed the most significant differences, while the comparison between AFC and AFM showed the least variation. We postulated that the transcriptome variations of swiftlets’ salivary glands are mainly due to dietary behavior and differences in genetics. Dietary behavior is presumed to influence the expression profiles of the salivary glands from AFC and AFM, as demonstrated in a previous study in broiler chickens that both feeding conditions and dietary manipulation significantly affect gene expression [40]. Moreover, in mice, it has been established that both genetic background and diet composition influence salivary gland gene expression [41]. Besides genetics and dietary behaviour, it seems that gender also influenced the regulation of genes in the salivary gland of EBN-producing swiftlets. Studies have reported the occurrence of sex-biased in gene expression profiles of avian and mammalian species [42]. However, the gender of the swiftlets probably has little effect on the expression profiles of the salivary glands, as 98% of the regulated genes have log2 fold changes that are equal or lesser than four.

NanoString technology was selected as an independent verification platform to determine the extent of correlation in gene expression between different salivary glands samples as the platform allows for highly multiplexed reactions and is able to generate an accurate signal capture as a digital readout [34]. Although NanoString and NGS-based analysis are robust assays for transcriptomic studies, NGS–based, RNA-sequencing detects differentially expressed transcripts by counting the sequencing library fragments that map to the exons of each gene and divide the count for each gene by a scaling factor based on the length of the exons [43]. The NanoString nCounter system is designed to detect nucleic acids based on unique probe hybridization chemistry without the involvement of any PCR amplification step. Each target molecule of interest was identified by the color code generated by the ordered fluorescent segments present on the reporter probe. A total of 33 genes from the NanoString results showed similar expression patterns when compared to their corresponding RNA-Seq. However, three genes showed contradicting patterns during the validation analysis. In addition, a low correlation of 0.54 was also detected in the expression profiles detected by both platforms. One possible reason for this variation is the sampling strategy adopted in this study, whereby in NGS analysis, both partially developed and well-developed salivary glands samples were pooled to create the reference transcript for the differential expression analysis, whereas in the NanoString system, only well-developed individual salivary gland samples were separately analyzed. Hence, the different sample batches and preparation methods might have caused the unwanted methodological bias. A previous study has also shown a poor correlation (r^2^) between transcriptome sequencing and NanoString analysis on formalin fixed paraffin-embedded breast tumor samples, which ranged from 0.59 to 0.72, due to variations in sample source and poor sample quality [44].

### Genes involved in salivary gland development

**Fig. 3:**
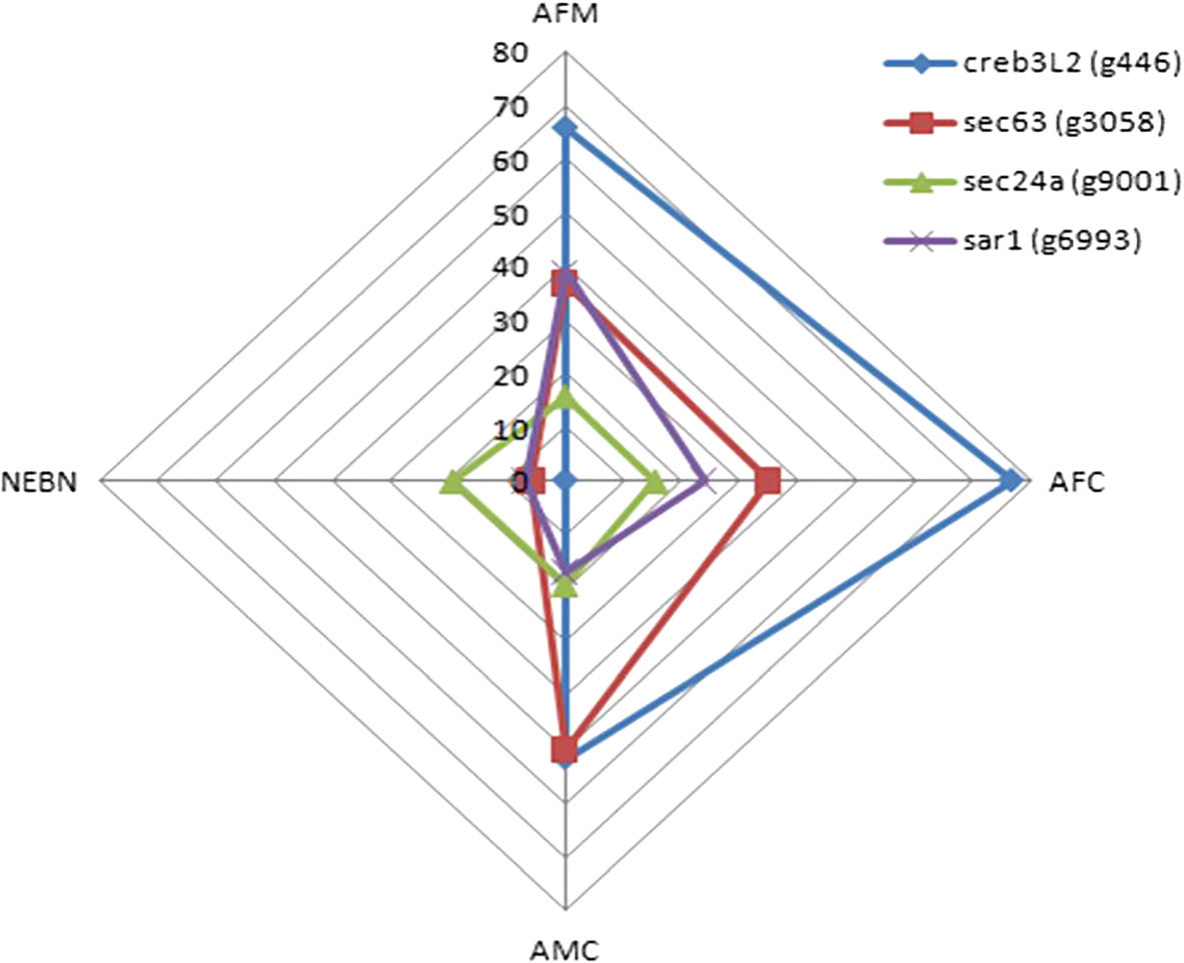
*CREB3L2* gene and cell type-specific secreted components genes (SPCGs). Radar draft representation of *CREB3L2* gene and cell type-specific secreted components genes (SPCGs) expression in various swiftlet groups; NEBN: Non-edible bird’s nest swift, AFM: *A. fuciphagus* from man-made house, AFC: *A. fuciphagus* from cave, AMC: *A. maximus* from cave

Salivary glands are remarkable organs. Despite their compact size they are able to produce the required volume of saliva via the interconnected network of secretory acini and ducts through a complex, epithelial-branching, morphogenesis process. Research on fly (*Drosophila melanogaster*) salivary glands identified both *CREBA* and *PAPS synthetase* genes as early and late markers of salivary gland development, respectively [45, 46]. A previous study on fly salivary glands showed that *CREBA* is a *Drosophila* homolog of the *CREB3* family of transcription factors which includes five different proteins in mammals, namely *CREB3*, *CREB3L1*, *CREB3L2*, *CREB3L3*, and *CREB3L4*. Each member of the Creb3-like family has a unique expression pattern in different organs and tissues [47]. Through RNA-Seq and gene expression analysis, differential expression analysis among different swiftlet species identified that expression of the *CREB3L2* gene was significantly regulated in EBN-producing swiftlets compared with AA (Fig. 3). Studies on the salivary glands *D. melanogaster* and *Aedes aegypti* revealed that *CREB3L2* and the expression of several important cell type-specific secreted component genes (SPCGs) are involved in salivary secretion and control of the saliva flow rate [45, 46]. SPCGs are genes that encode for protein machinery that targets and/or translocates proteins in the endoplasmic reticulum, regulates vesicle transport between the endoplasmic reticulum and Golgi, and cleaves the N-terminal signal sequence [47]. Several SPCGs were up-regulated in EBN-producing swiftlets compared to AA, including *SEC63*, *SEC24A*, and *SAR1*. The *SEC63* gene is an important regulatory gene involved in the translation of the endoplasmic reticulum, while both *SEC24A* and *SAR1* play an important role in exogenesis from the rough endoplasmic reticulum to the Golgi apparatus [48, 49].

In addition to *CREB3L2* and SPCGs, EBN-producing swiftlets also showed higher expression of the family of epidermal growth factor receptor (EGFR) genes compared with non-EBN-producing swiftlets. The EGFR family plays a vital role in mammalian development because it is involved in cell proliferation, migration, differentiation, survival, and apoptosis [50, 51] and it participates in the development and morphogenesis of salivary glands [52]. Multiple members of the epidermal growth factor (EGF) family of genes are expressed during salivary gland development, namely: *EGRF (ERBB1), ERBB2, ERBB3*, transforming growth factor-alpha (*TGF-a*), heparin binding EGF (*HBEGF*), and neuregulin (*NRG*) [53]. Furthermore, mutations in *EGRF, ERBB3* and *NRG1 type III* genes are also often associated with a reduction in salivary gland branching [54–56]. The relatively low expression of *CREB3L2*, SPCGs, and EGF receptor family genes in AA compared with EBN-producing swiftlets might restrict the development of the salivary glands in non-EBN-producing swiftlets and further hinder their ability to produce EBN.

The bone morphogenetic protein (BMP) signaling pathway and the mitogen-activated protein kinase (MAPK) signaling pathway were up-regulated in AFM compared to AFC (Table 4). BMPs are members of the transforming growth factor-beta (*TGF-β*) superfamily and are important during embryonic patterning and in the development of many different organ systems [57]. The BMP signaling pathway is closely associated with cell migration, proliferation, differentiation, and apoptosis. The BMP signaling pathway is important for the activation of SMAD-dependent (canonical) pathways as well as SMAD-independent (non-canonical) signaling pathways (*MAPK*, *PI3K/AKT*, *PKC*, *Rho-GTPases*), which are usually associated with cell proliferation and survival [58, 59]. Furthermore, *BMP7-*deficient mice have aberrant salivary glands morphogenesis with fewer end buds compared to wild-type controls [59–61], which suggests the involvement of BMP signaling pathway in acinar formation during salivary gland development. A previous study also suggested that multiple signaling pathways are tightly regulated in a spatiotemporal fashion during salivary gland development and morphogenesis [61].

### Genes involved in the composition of EBN

In Traditional Chinese Medicine (TCM), EBN is believed to promote the wellbeing of several organs and body systems, in addition to improving the immune system, strengthening bones, boosting metabolism, as well as having high antioxidant, anti-inflammatory, anti-aging, and anti-viral properties [4]. Various studies have characterized the composition and the therapeutic properties of EBN [10–12, 62–64]. However, the composition of EBN varies according to nest type, geographical factors, and harvesting period [62]. The fatty acid composition of EBN is highly correlated to environmental factors, such as the dietary pattern of the swiftlet and the availability of food at a specific location [63]. Swiftlet salivary glands are unique tissues with functions beyond the digestion of food and maintenance of the health of the oral cavity. Similarly, in certain animals for example arthropods, the salivary glands are equipped with cells that produce threads for the formation of webs, while in poisonous snakes, the salivary glands produce venomous saliva [61]. Among the major components isolated from EBN, EGF, glycoprotein, and sialic acid are used as indicators for the grading of EBN [63]. The results of the current study showed that *MGAT*, a gene essential for the biosynthesis of hybrid and complex N-acetylneuraminic acid, is highly expressed in EBN-producing swiftlets compared to AA*.* Further analysis indicated that AFC samples have the highest expression of this gene, followed by AFM, AMC, and lastly AA samples (Table 4). This finding is in conformity with that of a previous study that reported that non-EBN (grass-nest) contained a similar composition of essential monoses and EGF that is similar to EBN, but at a lower concentration [64]. The current study also suggested that white EBN produced by *A. fuciphagus* have the highest sialic acid and EGF contents, followed by black EBN produced by *A. maximus*, and lastly, grass-nest produced by non-EBN-producing swiftlets. Furthermore, gender-based analysis from the current study too indicated that *EDEM2*, a gene that is involved in trimming and biosynthesis of glycoproteins from the endoplasmic reticulum lumen [65], was significantly up-regulated in male AFC compared to female samples. A previous study on swiftlet nesting behaviour suggested that male swiftlets from man-made house contribute more to nest building compared to female swiftlets [66]. The current study also postulated that such phenomenon is generally because spermatogenesis is demanding less energy compared to oogenesis. Nevertheless, a future study on sex-biased gene expression profiling of swiftlet species from different habitats will be valuable to provide a comprehensive understanding of swiftlet nesting behaviour.

Generally, the quality of EBN is determined by the size, shape, origin, and color of the nest [9]. Currently, white EBN is considered the highest grade EBN and is commercialized at a premium price compared to black EBN [2]. One possible reason for this is that white EBN produced by *A. fuciphagus* contains higher concentrations of sialic acid and EGF [10, 62–64]. Nevertheless, the potential of black EBN should not be overlooked as our transcriptome analysis indicated that the alanine and aspartate metabolic pathways were up-regulated in salivary gland samples from AMC compared with AFC as the gene expression of both pyruvate dehydrogenase (*PDHB*) and glutamic-oxaloacetic transaminase (*GOT1*) was higher in samples from AMC compared to AFC (Table 2). *PDHB* is an important cofactor in amino acid biosynthesis and *GOT1* is a pyridoxal phosphate-dependent enzyme that plays a role in amino acid metabolism and the urea and tricarboxylic acid cycles [67]. A further in-depth study should be carried out to determine the composition and the medicinal values of black EBN.

## Conclusions

In conclusion, the transcriptomic profiling of salivary glands of different swiftlet species reveal differential expressions of candidate genes that are involved in salivary gland development and in the biosynthesis of various bioactive compounds found in EBN. Further functional characterization of these genes can provide insights into the mechanisms that regulate the production of saliva and the identification of markers that are unique to the origins of EBN.

## Additional files

Additional file 1: Table S1. Sampling locations. (DOCX 23 kb)

Additional file 2: Table S2. List of salivary gland samples selected for NGS-based transcriptome profiling. (DOCX 26 kb)

Additional file 3: Figure S1. Workflow of transcriptome of swiftlets salivary glands based on NGS pipelines and downstream bioinformatics analyses. (DOCX 556 kb)

Additional file 4: Table S3. CEGMA analysis readings of assembled combine reference transcript. (DOCX 23 kb)

## Abbreviations

BMP: Bone Morphogenetic Protein
CEGMA: Core Eukaryotic Genes Mapping Approach
EBN: Edible-bird’s Nest
EGFR: Epidermal Growth Factor Receptor
GO: Gene Ontology
HMMs: Hidden Markov Models
KASS: Automatic Annotation Server
KEGG: Kyoto Encyclopaedia of Genes and Genome
MAPK: Mitogen-activated Protein Kinases
NGS: Next-generation Sequencing
NR: Non-redundant
SRA: Sequence Read Archive
WEGO: Web Gene Ontology Annotation Plot
