# Supplementary material for: De novo transcriptome analysis shows differential expression of genes in salivary glands of edible bird’s nest producing swiftlets
Source: BMC Genomics. 2017 Jul 3;18:504. doi: 10.1186/s12864-017-3861-9 (PMC5496224; doi:10.1186/s12864-017-3861-9)
Supplement: Supplementary file 1 — Sampling locations. (DOCX 23 kb) [file 12864_2017_3861_MOESM1_ESM.docx]

**Table S1.** Sampling locations.

| Sampling location | Habitat | GPS coordination | Number of sample | Species |
| --- | --- | --- | --- | --- |
| *Bota Kanan,* Perak, West Malaysia | Man-made house | N 04^o^20.824’  E100^o^52.826’ | 5 | *Aerodramus fuciphagus* |
| *Serdang*, Selangor, West Malaysia | Man-made house | N 03^o^40.5’  E 101^o^42.8.7’ | 3 | *Aerodramus fuciphagus* |
| *Gomantong Cave*, Sandakan, Sabah, East Malaysia | Natural cave | N 05^o^31.46.5’  E 118^o^4’.29.6’ | 8 | *Aerodramus fuciphagus* |
| *Gomantong Cave*, Sandakan, Sabah, East Malaysia | Natural cave | N 05^o^31.46.5’  E118^o^4’.29.6’ | 8 | *Aerodramus maximus* |
| *FELDA,* Johor, West Malaysia | Wild | N 02^o^59.62.19’  E102^o^89’.69.16’ | 2 | *Apus affinis* |
