# Supplementary material for: De novo transcriptome analysis shows differential expression of genes in salivary glands of edible bird’s nest producing swiftlets
Source: BMC Genomics. 2017 Jul 3;18:504. doi: 10.1186/s12864-017-3861-9 (PMC5496224; doi:10.1186/s12864-017-3861-9)
Supplement: Supplementary file 2 — List of salivary gland samples selected for NGS-based transcriptome profiling. (DOCX 26 kb) [file 12864_2017_3861_MOESM2_ESM.docx]

**Table S2.** List of salivary gland samples used for NGS-based transcriptome profiling.

| No | Sample ID | Species | Sex | Habitat | RIN No. | Gland weight |
| --- | --- | --- | --- | --- | --- | --- |
| 1 | AFM 1 | *A.fuciphagus* | F | Man-made house | 7.5 | 0.042 |
| 2 | AFM 2 | *A.fuciphagus* | F | Man-made house | 7.3 | 0.023 |
| 3 | AFM 3 | *A.fuciphagus* | M | Man-made house | 7.7 | 0.038 |
| 4 | AFM 4 | *A.fuciphagus* | F | Man-made house | 8.1 | 0.073 |
| 5 | AFM 5 | *A.fuciphagus* | F | Man-made house | 7.6 | 0.060 |
| 6 | AFM 6 | *A.fuciphagus* | M | Man-made house | 9.2 | 0.084 |
| 7 | AFM 7 | *A.fuciphagus* | M | Man-made house | 8.1 | 0.084 |
| 8 | AFM 8 | *A.fuciphagus* | M | Man-made house | 9.2 | 0.051 |
| 1 | AFC 1 | *A.fuciphagus* | M | Natural cave | 9.3 | 0.039 |
| 2 | AFC 2 | *A.fuciphagus* | F | Natural cave | 9.3 | 0.016 |
| 3 | AFC 3 | *A.fuciphagus* | F | Natural cave | 7.9 | 0.057 |
| 4 | AFC 4 | *A.fuciphagus* | F | Natural cave | 7.7 | 0.064 |
| 5 | AFC 5 | *A.fuciphagus* | M | Natural cave | 8.2 | 0.057 |
| 6 | AFC 6 | *A.fuciphagus* | M | Natural cave | 7.9 | 0.063 |
| 7 | AFC 7 | *A.fuciphagus* | M | Natural cave | 6.6 | 0.098 |
| 8 | AFC 8 | *A.fuciphagus* | F | Natural cave | 7.2 | 0.093 |
| 1 | AMC 1 | *A.maximus* | M | Natural cave | 7.9 | 0.034 |
| 2 | AMC 2 | *A.maximus* | F | Natural cave | 9.3 | 0.025 |
| 3 | AMC 3 | *A.maximus* | M | Natural cave | 8.3 | 0.094 |
| 4 | AMC 4 | *A.maximus* | F | Natural cave | 8.1 | 0.059 |
| 5 | AMC 5 | *A.maximus* | M | Natural cave | 7.9 | 0.073 |
| 6 | AMC 6 | *A.maximus* | F | Natural cave | 7.0 | 0.059 |
| 7 | AMC 7 | *A.maximus* | F | Natural cave | 7.9 | 0.051 |
| 8 | AMC 8 | *A.maximus* | M | Natural cave | 8.5 | 0.054 |
| 1 | AA 1 | *Apus affinis* | F | Wild | 7.9 | 0.044 |
| 2 | AA 2 | *Apus affinis* | M | Wild | 7.8 | 0.049 |
