# Supplementary figures and images for: De novo transcriptome analysis shows differential expression of genes in salivary glands of edible bird’s nest producing swiftlets
Source: BMC Genomics. 2017 Jul 3;18:504. doi: 10.1186/s12864-017-3861-9 (PMC5496224; doi:10.1186/s12864-017-3861-9)

**
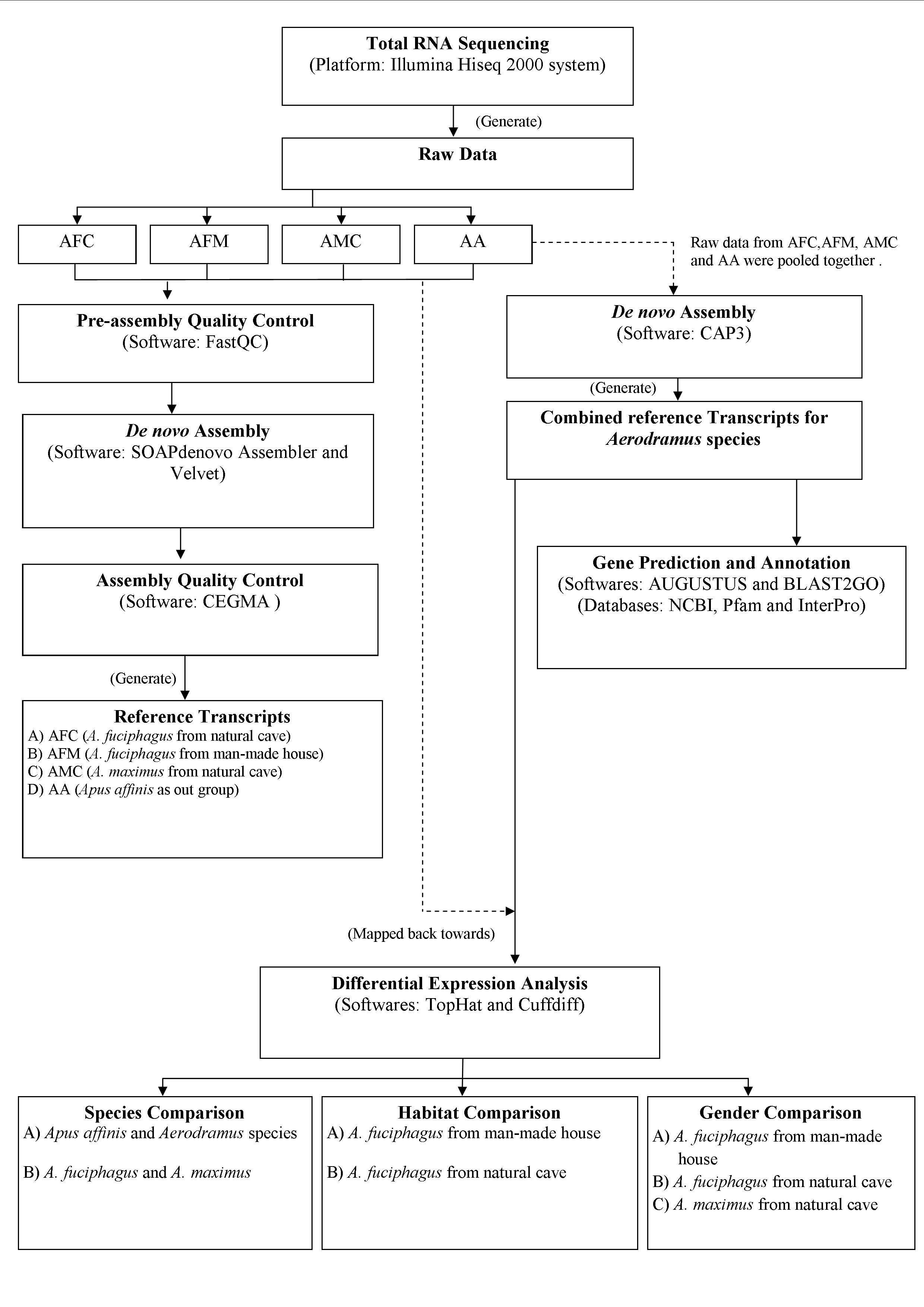
**

Supplement: Supplementary file 3 — Workflow of transcriptome of swiftlets salivary glands based on NGS pipelines and downstream bioinformatics analyses. (DOCX 556 kb) [file 12864_2017_3861_MOESM3_ESM.docx]
