# Supplementary material for: De novo transcriptome analysis shows differential expression of genes in salivary glands of edible bird’s nest producing swiftlets
Source: BMC Genomics. 2017 Jul 3;18:504. doi: 10.1186/s12864-017-3861-9 (PMC5496224; doi:10.1186/s12864-017-3861-9)
Supplement: Supplementary file 4 — CEGMA analysis readings of assembled combine reference transcript. (DOCX 23 kb) [file 12864_2017_3861_MOESM4_ESM.docx]

**Table S3.** CEGMA analysis readings of assembled combine reference transcript.

|  | Prots | % Completeness | Total | Average | % Ortho |
| --- | --- | --- | --- | --- | --- |
| Complete | 143 | 57.66 | 401 | 2.8 | 72.03 |
| Group 1 | 23 | 34.85 | 71 | 3.09 | 73.91 |
| Group 2 | 27 | 48.21 | 65 | 2.41 | 66.67 |
| Group 3 | 43 | 70.49 | 121 | 2.81 | 76.74 |
| Group 4 | 50 | 76.92 | 144 | 2.88 | 70.00 |
| Partial | 185 | 74.60 | 663 | 3.58 | 86.49 |
| Group 1 | 40 | 60.61 | 152 | 3.80 | 95.00 |
| Group 2 | 35 | 62.50 | 114 | 3.26 | 82.86 |
| Group 3 | 50 | 81.97 | 184 | 3.68 | 84.00 |
| Group 4 | 60 | 92.31 | 213 | 3.55 | 85.00 |

Key:

Prots = number of 248 ultra-conserved CEGs present in genome

%Completeness = percentage of 248 ultra-conserved CEGs present

Total = total number of CEGs present including putative ortholog

Average = average number of orthologs per CEG

%Ortho = percentage of detected CEGS that have more than 1 ortholog
